# Multidrug-Resistant Typhoid Fever Outbreak in Travelers Returning from Bangladesh

**DOI:** 10.3201/eid1312.070422

**Published:** 2007-12

**Authors:** Yasuyuki Kato, Makiko Fukayama, Takuya Adachi, Akifumi Imamura, Takafumi Tsunoda, Naohide Takayama, Masayoshi Negishi, Kenji Ohnishi, Hiroko Sagara

**Affiliations:** * Tokyo Metropolitan Bokutoh Hospital, Tokyo, Japan; † Tokyo Metropolitan Toshima Hospital, Tokyo, Japan; ‡ Yokohama Municipal Citizen’s Hospital, Yokohama, Japan; § Tokyo Metropolitan Komagome Hospital, Tokyo, Japan; ¶ Tokyo Metropolitan Ebara Hospital, Tokyo, Japan; 1 Current affiliation: International Medical Center of Japan, Tokyo, Japan; 2 Current affiliation: Tokorozawa Loyal Hospital, Saitama, Japan; 3 Current affiliation: Japan International Cooperation Agency, Tokyo, Japan; 4 Current affiliation: Negishi Medical Clinic, Tokyo, Japan

**Keywords:** Typhoid fever, food poisoning, Salmonella typhi, disease outbreaks, drug therapy, adolescent, travel, case report, letter

**To the Editor:** Enteric fever (typhoid and paratyphoid fever) is a systemic infection caused by several *Salmonella enterica* serotypes including *S*. Typhi and *S*. Paratyphi A. The Indian subcontinent, which has the highest incidence of the disease worldwide, is also an epicenter of enteric fever caused by multidrug-resistant (MDR; resistant to chloramphenicol, ampicillin, and trimethoprim-sulfamethoxazole) and nalidixic acid–resistant (NAR) strains, i.e., strains with decreased susceptibility to ciprofloxacin (*1*–*3*). A total of 57% of *S*. Typhi strains isolated at a referral center in Dhaka, Bangladesh, in 2005 were MDR and NAR (*4*).

More than 80% of 442 enteric fever cases reported in Japan during 2001–2004 were imported (*5*). Most Japanese persons, especially the younger generation, are not immune to enteric fever as are persons living in other industrialized countries.

Although the proportion of enteric fever cases related to international travel has increased in industrialized countries, few outbreaks of enteric fever have been reported in travelers (*6*,*7*). We describe an outbreak of MDR and NAR typhoid fever in young Japanese travelers returning from Bangladesh. This outbreak highlights the need for standard treatments for MDR and NAR enteric fever.

Ten Japanese junior and senior high school students living in the Tokyo metropolitan area took part in a 9-day study tour to Dhaka in March–April, 2004. They were escorted by 2 Japanese college students and a 28-year-old Japanese instructor. The 13 participants returned to Japan on April 4, 2004. The purpose of the study tour was to acquire knowledge about street children in Dhaka. The students stayed at a guesthouse and visited orphanages in the city. The itinerary included a visit to a local home, where the family served them a meal. They shared all their meals during the tour.

Fever and diarrhea developed in 2 participants on April 3 and 5, and these symptoms were later shown to be caused by shigellosis. On April 19, the index patient became febrile. From that date until April 28, there were 6 confirmed and 2 probable typhoid fever cases reported in the 13 tour participants, resulting in an attack rate of 62%. The median age of the patients was 17 years (range 12–28 years); 5 patients were female. No other cases of typhoid fever were reported in that period in Japan.

All 6 *S*. Typhi isolates were Vi-phage type E9. These isolates were also MDR and NAR, and the MIC for ciprofloxacin for the 6 isolates was 0.38 μg/mL. It was strongly suspected that a single-point exposure to *S*. Typhi occurred in the tour participants during their stay in Bangladesh and caused this exceptional outbreak. None of the participants had received a typhoid vaccination.

The 8 patients were admitted to 5 hospitals in the Tokyo metropolitan area. Four different antimicrobial drug regimens were used on the basis of the age of the patients and the hospital in which each patient was hospitalized (Table). Four patients at 2 hospitals who received fluoroquinolone monotherapies were given other regimens on days 4–6 of treatment because of concern of treatment failure. The median fever clearance time was 6 days (range 3–12 days). No complications occurred during any of the treatment regimens. Although a relapse occurred 15 days after completion of treatment in the oldest patient, who had received cefotaxime and oral tosufloxacin, retreatment cured the infection without fecal carriage.

**Table:** Characteristics of 8 case-patients with typhoid fever, Bangladesh, 2004*

Case- patient†	Age, y/ sex	Date of onset	Vi-phage type	Ciprofloxacin MIC, μg/mL	Cefotaxime MIC, μg/mL	Treatment‡	FCT, d
1C	28/F	Apr 19	E9	0.38	0.094	Ciprofloxacin 500 mg 2× a day for 3 d, cefotaxime 1 g every 12 h + tosulfoxacin 300 mg 2× a day for 11 d	4§
2C	17/F	Apr 20	E9	0.38	0.094	Levofloxacin 200 mg 2× a day for 14 d	6
3C	17/F	Apr 21	E9	0.38	0.094	Ciprofloxacin 500 mg 2× a day for 3 d, cefotaxime 1 g every 12 h + tosulfoxacin 300 mg 2× a day for 11 d	3
4P	19/F	Apr 21	NA	NA	NA	Levofloxacin 200 mg 2× a day for 3 d, cefotaxime 1 g every 12 h + tosulfoxacin 300 mg 2× a day for 13 d	12
5C	12/M	Apr 22	E9	0.38	0.094	Azithromycin 1 g for 1 d, 500 mg a day for 2 d; norfloxacin 250 mg 3× a day for 11 d	7
6C	16/F	Apr 23	E9	0.38	0.094	Levofloxacin 500 mg a day for 14 d	5
7C	19/M	Apr 23	E9	0.38	0.064	Ciprofloxacin 500 mg 2× a day for 5 d, ceftriaxone 2 g every 12 h for 16 d	6
8P	15/M	Apr 28	NA	NA	NA	Levofloxacin 200 mg 2× a day for 18 d	7

*MICs were determined by E-test (AB Biodisk, Solna, Sweden). MICs of chloramphenicol, ampicillin, trimethoprim-sulfamethoxazole, and nalidixic acid were >256 μg/mL, >256 μg/mL, >32 μg/mL, and >256 μg/mL, respectively. FCT, fever clearance time (time from the start of treatment until the body temperature reached 37.5°C and remained at 37.5°C for 48 h); NA:, not available. †C, confirmed case, i.e., a patient with fever (>38°C) for >3 d and a laboratory-confirmed positive blood culture for *Salmonella enterica* serotype Typhi; P, probable case, i.e., a patient with fever (>38°C) for >3 d without isolation of *S*. Typhi. ‡Daily dosages are shown. All fluoroquinolones were given orally. Tosufloxacin is a fluoroquinolone with properties similar to those of levofloxacin. §Fever relapsed 15 d after completion of treatment. Retreatment with tosufloxacin, 600 mg/d for 14 d, was successful.

The high attack rate may reflect the high sensitivity of adolescents to typhoid fever and the high level of bacterial contamination in food the participants had eaten during travel (*2*). Although the meal at the private home was suspected as the source of infection, we could not determine the exact cause of this outbreak.

The optimum treatment for MDR and NAR enteric fever has not yet been established. A third-generation cephalosporin or high doses of fluoroquinolones (e.g., ciprofloxacin, 20 mg/kg/day or levofloxacin, 10 mg/kg/day) for 10–14 days are the drugs of choice (*1*,*2*). Azithromycin is also a promising agent (*8*). However, for any of the regimens, the mean fever clearance times are relatively long (≈7 days), and the relapse rates are high (*1*). Although all 6 isolates showed reduced susceptibility to ciprofloxacin, a long course (14 days) of fluoroquinolones was still effective in this outbreak. However, clinicians should be aware of treatment failure in MDR and NAR enteric fever (*3*). The combination therapy of cefotaxime and a fluoroquinolone used in 3 patients has not shown greater efficacy than monotherapies. In fact, 1 patient who received this combination therapy experienced a relapse.
